# A Conservative Replacement in the Transmembrane Domain of SARS-CoV-2 ORF7a as a Putative Risk Factor in COVID-19

**DOI:** 10.3390/biology10121276

**Published:** 2021-12-05

**Authors:** Andrei Lobiuc, Daniel Șterbuleac, Olga Sturdza, Mihai Dimian, Mihai Covasa

**Affiliations:** 1 Department of Biomedical Sciences, College of Medicine and Biological Sciences, “Ștefan cel Mare” University of Suceava, Str. Universității 13, 720229 Suceava, Romania; andrei.lobiuc@usm.ro (A.L.); daniel.sterbuleac@usm.ro (D.Ș.); olga.caliman-sturdza@usm.ro (O.S.); 2 Division of Infectious Diseases, Suceava County Regional Emergency Hospital, 720229 Suceava, Romania; 3 Integrated Center for Research, Development and Innovation in Advanced Materials, Nanotechnologies and Distributed Systems for Fabrication and Control (MANSiD), “Ștefan cel Mare” University of Suceava, Str. Universității 13, 720229 Suceava, Romania; dimian@usm.ro; 4 Department of Computers, Electronics and Automation, “Ștefan cel Mare” University of Suceava, 720229 Suceava, Romania

**Keywords:** molecular dynamics, ORF7a, protein modeling, RMSF, viral mutation

## Abstract

**Simple Summary:**

The pathogenicity and transmissibility of the COVID-19 pandemic causative agent, the SARS-CoV-2 virus, is related to the functions of the proteins synthesized intracellularly, as guided by viral RNA. It is vitally important to accurately pinpoint novel variants of concern of the SARS-CoV-2 virus, in order to understand the molecular features of novel mutations and manage the on-going battle against the COVID-19 pandemic. We focused on A105V mutation in the ORF7a accessory protein. Sequencing and clinical data showed that this mutation is associated with increased severity and lethality in a group of Romanian patients, despite a lower viral copy number and a lower number of associated comorbidities. This effect is primarily due to increased protein stability through allosteric effects as shown by molecular dynamics analyses. This behavior manifests especially among residues 39–56, and the ones adjacent to 26–30 loop, placed in direct contact with potential interaction partners. Together, the results provide novel insights into the role of ORF7a in the pathogenicity of SARS-CoV-2.

**Abstract:**

The ongoing COVID-19 pandemic follows an unpredictable evolution, driven by both host-related factors such as mobility, vaccination status, and comorbidities and by pathogen-related ones. The pathogenicity of its causative agent, SARS-CoV-2 virus, relates to the functions of the proteins synthesized intracellularly, as guided by viral RNA. These functions are constantly altered through mutations resulting in increased virulence, infectivity, and antibody-evasion abilities. Well-characterized mutations in the spike protein, such as D614G, N439K, Δ69–70, E484K, or N501Y, are currently defining specific variants; however, some less studied mutations outside the spike region, such as p. 3691 in NSP6, p. 9659 in ORF-10, 8782C > T in ORF-1ab, or 28144T > C in ORF-8, have been proposed for altering SARS-CoV-2 virulence and pathogenicity. Therefore, in this study, we focused on A105V mutation of SARS-CoV-2 ORF7a accessory protein, which has been associated with severe COVID-19 clinical manifestation. Molecular dynamics and computational structural analyses revealed that this mutation differentially alters ORF7a dynamics, suggesting a gain-of-function role that may explain its role in the severe form of COVID-19 disease.

## 1. Introduction

Severe acute respiratory syndrome (SARS) is a novel disease caused by two coronaviruses, namely SARS-CoV and SARS-CoV-2, that emerged in 2002 and 2019, respectively [1]. The disease caused by the SARS-CoV outbreak had a very high fatality rate (~10%) with approximately 8000 cases worldwide [2]. Comparatively, SARS-CoV-2 is responsible for one of the worst pandemics in modern history, with over 246 million cases worldwide and in excess of 5 million deceased as of November, 3rd, 2021 [3]. The fatality rate of the current pandemic is much lower (~2%), but the features of the unusually high transmissibility of SARS-CoV-2 are far from being understood. SARS-CoV-2 also induces a very broad clinical spectrum, ranging from asymptomatic and mild illness to the most severe forms [4].

The SARS-CoV-2 genome is approximately 29.9 kb in length, with 12 open reading frames (ORFs). It encodes two large polyproteins, four structural proteins, and eight accessory proteins [5]. The two large polyproteins are ORF1a and ORF1b, which proteolytically cleave to form 16 non-structural proteins (NSPs) [6]. Open reading frame member 7a of SARS-CoV, abbreviated ORF7a, is an accessory protein expressed in both SARS-CoV and SARS-CoV-2 viruses. Significant insights into its role in SARS-CoV disease-inducing mechanisms were mostly identified through on-going efforts following the 2003 pandemic. SARS-CoV ORF7a is located intracellularly in the infected cells, localizing in the Golgi apparatus and on the cell surface [7]. It is 122 residues long, has a molecular weight of 5.5 kDa, and its roles are related to augmenting SARS-CoV virulence. The putative mechanisms surrounding its disease-enhancing effects were linked to the induction of caspase-dependent apoptosis, as shown in several cell lines infected with SARS-CoV [8]. ORF7a is also involved in inhibition of protein synthesis and in cell cycle blockage and has pro-inflammatory actions, interfering with the normal host cellular environment [9,10].

While information on the putative roles of SARS-CoV-2 ORF7a is starting to emerge, its cellular roles are far from being understood. The sequence alignment of SARS-CoV and SARS-CoV-2 ORF7a proteins exhibits 85.2% identity and 95.9% similarity [6], while overlapping of the 3D structures of their extracellular globular domains reveals a root mean square deviation (RMSD) of only 0.411 Å. However, different functional roles have been observed for ORF7a of SARS-CoV-2, compared to SARS-CoV. For example, SARS-CoV-2 ORF7a binds to CD14+ monocytes in human peripheral blood with much higher affinity than SARS-CoV ORF7a [11]. This enacts altered receptor expression on CD14^+^ monocytes, leading ultimately to accumulation of CD14+ monocytes in patients with severe forms of COVID-19. This forms the basis of a likely mechanism through which ORF7a mediates the potentially fatal cytokine storm progression in COVID-19 patients, indicating that ORF7a may be a key viral factor for severity [11]. In addition, computational structural investigations have shown that SARS-CoV-2 ORF7a possesses the structural determinants required for binding to T lymphocytes and macrophages [12]. SARS-CoV-2 ORF7a is also linked to viral transmission and pathogenesis through inhibition of interferon signaling [13,14]. Several studies have reported deletions in the ORF7a gene in SARS-CoV-2 isolates, but were unable to place them in the clinical context [15,16,17,18,19]. However, in vitro deletion of ORF7a gene reduces replication of the synthetic virus [20], suggesting an important role of ORF7a in disease severity.

We have previously sequenced the SARS-CoV-2 viral genome isolated from a group of 62 Romanian patients during first COVID-19 outbreak and identified several mutations, one of which occurred at position 27,707 (C27707T) which leads to a substitution of alanine to valine (A105V) in the transmembrane domain of SARS-CoV-2 ORF7a [21]. This mutation has been associated with a more severe clinical outcome and a high death rate, thus directly linking ORF7a mutation with disease severity [21]. Based on this and given that structural information about SARS-CoV-2 ORF7a is available, we conducted a structural molecular dynamics analysis, which revealed how this mutation differentially alters ORF7a dynamics, suggesting a gain-of-function role.

## 2. Materials and Methods

### 2.1. Sample Collection

Viral RNA was collected from patients hospitalized in Suceava County Regional Hospital during the April–June 2020 period. Patients signed informed consent for data access, and the study was approved by the University of Suceava Research Ethics Committee (protocol number 11733/14.07.2020). Criteria for patient selection included age, sex, severity of the disease, number of days in hospital, and existing comorbidities. Samples were collected by nasopharyngeal swabs from patients presenting with COVID-19-like symptoms. Clinical, epidemiological, and demographic data were taken from patients’ medical records. These patients were among the first cohort identified with A105V mutation during the largest COVID-19 outbreak in Northeastern Romania and used to track the origin of virus introduction in Romania [21].

### 2.2. Sample Preparation and Sequencing

RNA extraction was performed using Bioneer AccuPrep^®^ Viral RNA Extraction Kit. RNA extracts were evaluated for RNA quantity (Qubit ssRNA HS kit, Invitrogen Inc., Eugene, OR, USA) and for viral copy numbers (TaqMan 2019-nCoV Assay Kit v1, Applied Biosystems, Pleasanton, CA, USA). SARS-CoV-2 samples were selected for analysis based on quantity and quality of viral RNA. RNA (100 ng) was reverse transcribed using SuperScript™ VILO™ cDNA Synthesis Kit (Invitrogen, Carlsbad, CA, USA), according to product protocol. Targets for sequencing were obtained based on Ion AmpliSeq™ SARS-CoV-2 Panel (ThermoFisher Scientific, Waltham, MA, USA). Library preparation was made using Ion AmpliSeq™ Library Kit Plus (ThermoFisher Scientific, Carlsbad, CA, USA), then libraries were loaded on sequencing chips using Ion Chef equipment. Next-generation sequencing was performed on Ion S5 Gene Studio, using Ion Torrent 540 chips.

### 2.3. Sequencing Data Processing, Data Availability, and Mutation Analysis

Sequencing reads were mapped and assembled using the Iterative Refinement Meta Assembler (IRMA), after which variants were called using Torrent VariantCaller plugin, referenced to the Wuhan SARS-CoV-2 sequence and annotated using SnpEff plugin. Sequences were uploaded in GISAID database on 11 July 2020 and 16 July 2020. Identified mutations were tabulated and analyzed for frequency and recurrence. In order to discern from intrapopulation variants and to focus on potentially transmissible ones, only mutations with >99% frequency in sequencing reads were further processed, and those present in >5% of the samples were selected.

### 2.4. ORF7a Protein Modeling

The crystal structure of the intracellular part of SARS-CoV-2 ORF7a was identified and deposited in March 2020 in the Protein Data Bank (PDB) under the accession number 6W37 [22]. It spans 67 amino acids (E16-V82), while lacking other segments of the entire 121-amino-acids-long full protein, including the easy-to-model helical transmembrane segment (L96-E116). Thus, structural information for ORF7a is sufficient to generate a reliable model of the full protein. Such a model had already been generated by the widely used Zhang Lab’s I-TASSER algorithms [23] and included in the “Genome-wide structure and function modeling of SARS-Cov-2” [24], from where it was retrieved. Prior to sending the model to molecular dynamics (MD) simulations, the transmembrane segment had been slightly rotated and directed apart from the core of the protein. The transmembrane domain and an adjacent loop (together comprising residues 97–121) were selected and directed apart from the protein core using the Movement Mouse Mode function (selection only) and visualized in Chimera 1.15 [25]. The transformation was performed without disrupting or creating any new residue−residue interaction, as seen in Figure S1. This was performed to correct the spatial arrangement of the protein core, the transmembrane segment, and the linker between them, which would have otherwise, upon insertion into the membrane, placed the protein core in contact with the lipid membrane instead of water. The protein coordinates of the final model are available upon request.

### 2.5. Molecular Dynamics (MD) Simulation Protocols and Analysis

To study the dynamics of ORF7a wild-type (WT) and its mutations, MD systems were built using the interactive CHARMM-GUI Membrane Builder [26]. The bilayer patch size was set at 62 Å × 62 Å, comprised entirely of phosphatidylcholine (POPC) lipid molecules. The transmembrane segment’s residues 101 and 112 provided the two spatial references needed for proper membrane alignment, using the “Align a Vector (Two Atoms) Along Z” option in Step 3 of the on-line Membrane Builder protocol. Other parameters were left to their default values. Ion concentration (KCl salt) was kept at 0.15 M and the simulation box was filled, top and bottom, with water molecules, providing at least 17.5 Å spacing between the protein and the cuboid limits. The CHARMM36m force field was employed [27]. Three systems were created: one for WT ORF7a and two for mutants A105V and A106V, respectively, the latter included to act as a second control to the computational study design. The mutations were introduced in Step 1 of the Membrane Builder protocol, as well as the disulfide links between residues 23–58 and 35–67.

The full MD systems comprised ~40,000 atoms. After the fully built MD systems were retrieved, the time-tested, seven-step protocol provided by CHARMM-GUI was employed. The first six steps were run as-is, to properly equilibrate the system, while the production simulation (7th step) spanned 30 ns of fully relaxed atomistic motion. All MD simulations were carried out with NAMD Alpha 3.0 [28], and each simulation was replicated six times. Analyses of MD trajectories and plots of the root-mean-square fluctuation (RMSF) were obtained with Visual Molecular Dynamics (VMD) v. 1.9.3 [29]. RMSF calculations were performed solely on alpha carbons, using an in-house VMD script. Prior to RMSF calculation, the transmembrane domain alpha carbons (residues 95–115) were aligned over all frames of simulation, using the **Align** function of the RMSD Visualizer Tool. The F-test was employed to determine per-residue differences in variance. Interaction energy decomposition was performed using the NAMDEnergy plug-in from VMD, calling NAMD 2.12 (multicore version) for actual calculations.

## 3. Results

### 3.1. Clinical Data and Putative Disease-Enhancing Role of ORF7a A105V

Next Generation Sequencing was performed on naso-pharyngeal samples collected from patients hospitalized with COVID-19 at Suceava County Emergency hospital during the first COVID-19 outbreak. This hospital was also the origin of the first and largest COVID-19 outbreak in Romania, in March 2020.

Out of 62 patients with viral sequences analyzed, 17 (27.5%) were infected with A105V recurrent mutation (average frequency in assembled reads 99.1%). A summary of clinical and non-clinical data is presented in Table 1. Patients infected with mutated SARS-CoV-2 ORF7a A105V exhibited twice the death rate when compared with the wild-type SARS-CoV-2 group. However, comparative clinical data showed that patients carrying viral RNA coding for ORF7a A105V exhibited an unusual clinical pattern characterized by both a lower number of associated comorbidities and lower viral copy number (based on PCR cycle threshold, Table 1). As such, half of patients in the WT group had at least one comorbidity, while only 35% in the mutated group presented with comorbidities. The number of patients with hypertension or obesity was approximately half in the mutant compared to WT-infected groups. At the same time, the mean viral load was more than double in patients infected with WT variant, while the median was 10,000 vs. 2500 (four times higher) in WT vs. A105V variant, respectively (data not shown).

**Figure 1:**
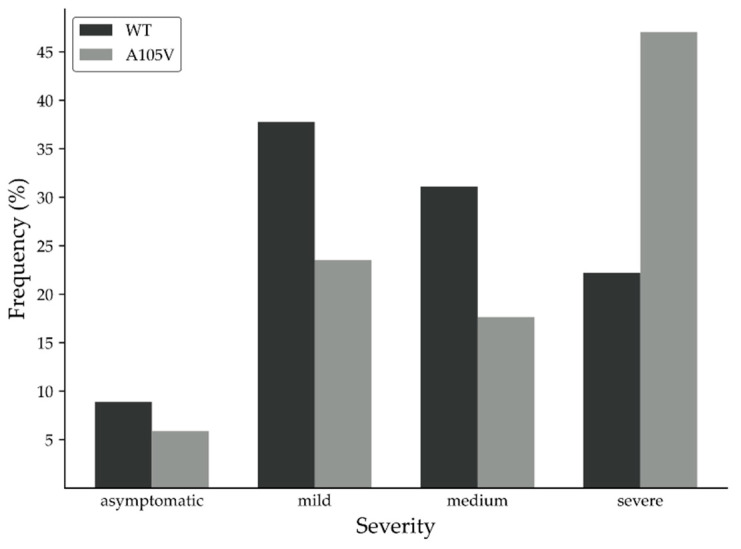
Disease severity in COVID-19 patients infected with wild-type (WT) and ORF7a A105V.

Despite their higher viral load and number of comorbidities, patients infected with WT ORF7a mutation showed, on average, a milder degree of symptoms (Figure 1). Approximately 47% of patients infected with ORF7a A105V variant developed a severe form of the disease, compared to only 22.2% infected with non-mutant variant. Asymptomatic and mild effects accounted for 46.5% of patients infected with WT variant, compared to 29.3% in the A105V group. Additionally, C-reactive protein (CRP) levels were significantly higher (9.7 mg/dL) in patients infected with the A105V compared to control (5.6 mg/dL), indicating a higher inflammation status.

**Figure 2:**
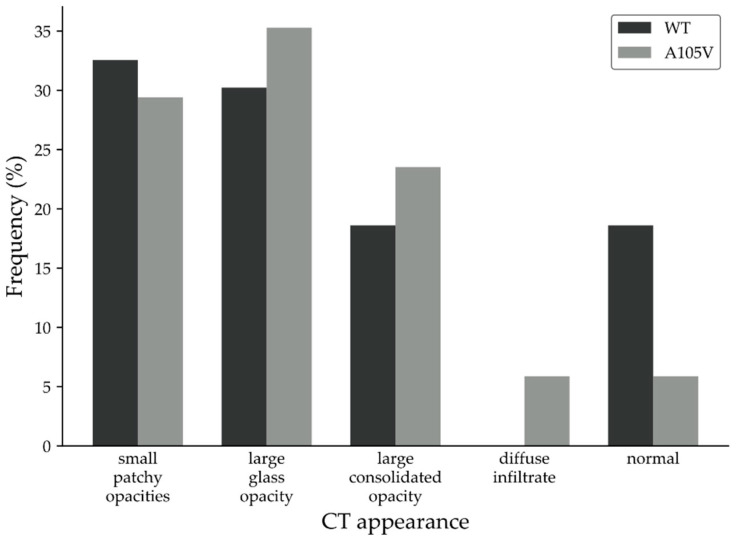
CT lung scans of patients infected with WT and ORF7a A105V.

Analyses of lung CT appearance correlated well with disease severity. Overall, lung CT scans of A105V patients showed increased severity compared with controls, with large glass opacities (35% vs. 30%), large consolidated opacities (23% vs. 18%), and diffuse infiltrates (5% vs. 0%) (Figure 2).

Comorbidity patient data showed that three, out of four, deceased patients due to COVID-19 in the WT group had associated comorbidities while two, out of three, patients who died due to infection with SARS-CoV-2 ORF7a A105V had no associated comorbidities.

### 3.2. Structural Dynamics of Mutant and Native ORF7a Proteins

In an attempt to validate the clinical outcome associated with ORF7a mutation, we sought to evaluate the behavior of the mutant protein under the computational structural biology lens. Thus, we determined whether A105V mutation alters protein dynamics, which may be linked to changes in ORF7a interaction landscape, by performing molecular dynamics (MD) simulations on three different proteins: the WT ORF7a, A105V ORF7a, and A106V ORF7a, with the first and the last as controls.

**Figure 3:**
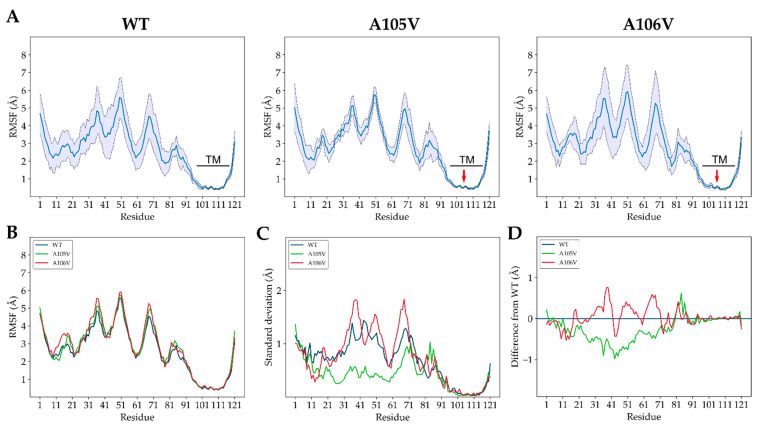
Results from the MD simulations of WT and mutant ORF7a. (**A**) Average per-residue RMSF of the three ORF7a models, WT, A105V, and A106V mutations, during MD simulations. Values are presented as mean (blue line) +/− standard deviation (thin dashed lines) over the course of the six replicas; the transmembrane (TM) residues’ localization (black line) and mutation location (red arrow) are also included. (**B**) Overlay of the average per-residue RMSF of the three ORF7a models. (**C**) Overlay of the standard deviation per-residue in the six simulations. (**D**) Overlay of the standard deviation from simulations of mutant ORF7a, relative to WT simulation (blue baseline).

Figure 3A presents the average RMSF over the course of 30 ns production runs (averages are performed over six different replications). The comparative examination of the three simulated proteins, based on the standard deviation calculated from the six replications (per-residue basis), showed that A105V mutation behaved differently compared to controls. Specifically, during MD simulations, the RMSF of the mutant protein showed a remarkable small standard deviation in the core of the protein, when compared with the two controls (residues 21–71, Figure 3A). An overlay of the three RMSF plots (Figure 3B) shows highly similar RMSF for all three simulations. However, visual representations of the absolute RMSF standard deviations per-residue revealed higher values for core residues (21–71) for both WT (Figure 3C) and A106V (Figure 3D) controls, in contrast with A105V. The standard deviation of the A106V RMSF simulation in the protein core displayed high variability, with both negative and positive values, relative to WT (Figure 3D, red line). However, the standard deviation of the RMSF in A105V mutant simulations was overall consistently smaller within the same region, compared to both WT and A105V (Figure 3D, green line). The calculated means (SE) of the standard deviations in the 21–71 region accurately reflect the observed effects (WT: 0.99 ± 0.03, A105V: 0.42 ± 0.01, A106V: 1.14 ± 0.06).

**Figure 4:**
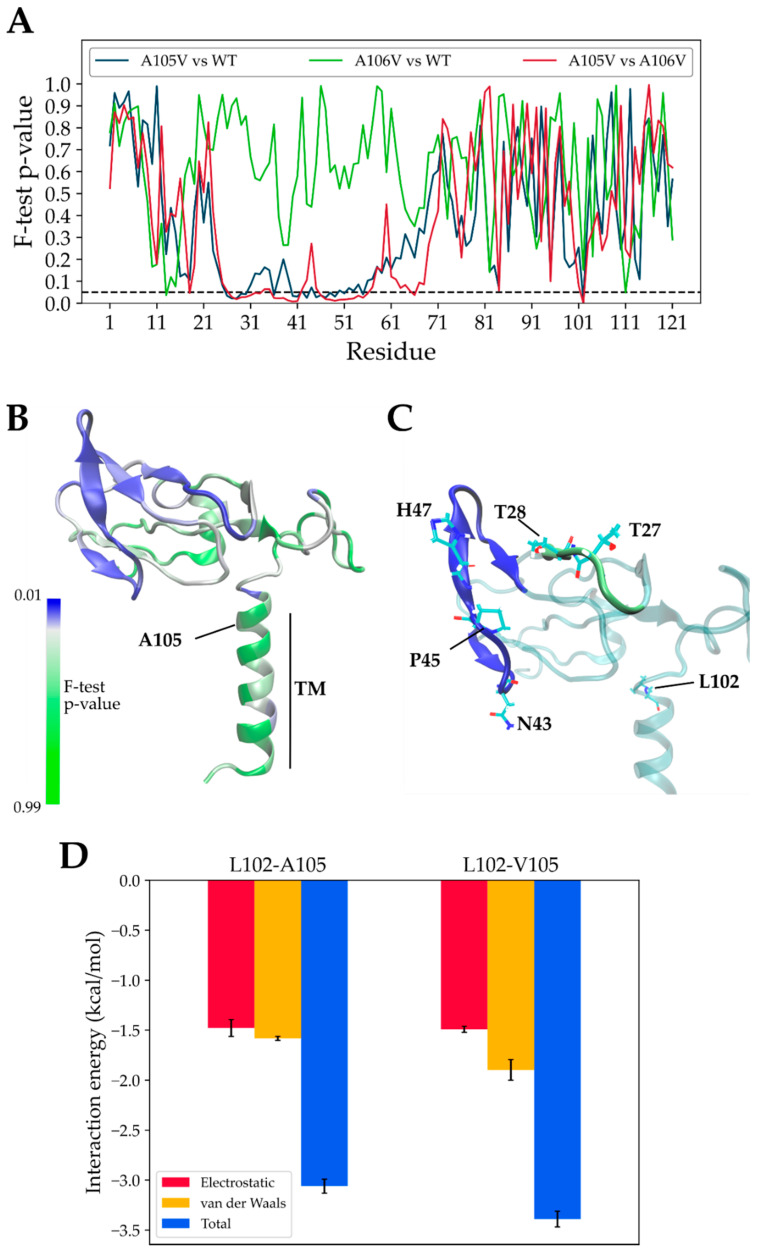
(**A**) The per-residue *p*-values retrieved from the three pair-wise F-tests of RMSF variance; the dashed horizontal line indicates the standard *p* = 0.05 reference. (**B**) Structure of the ORF7a model used in the simulation, indicating the position of A105 residue and the transmembrane (TM) helix; residues are colored based on the *p*-values obtained from A105V vs. wild-type F-tests for variance. (**C**) Location of six residues with *p* < 0.03 (depicted as light blue sticks) and of the two regions with very low F-test *p*-values (26–30, solid green ribbon; 39–56, solid blue ribbon); other ribbons are depicted in light transparent blue. (**D**) Decomposition of average interaction energies between residues in positions 102 and 105 during the six WT and A105V MD simulations (mean +/− standard error).

The overall results from the MD comparative analyses suggest that the A105V mutation induces a very specific change in the dynamic behavior of the mutated ORF7a protein. The low standard deviation of the RMSF (for A105V simulations) was due to similar protein dynamics in each of the six replica simulations. In the control groups, this was not the case, as the replicas diverged with respect to protein motion due to the stochastic nature of the MD algorithm. The A105V ORF7a assumed a much higher conformational stability compared to both wild-type and the A106V control, thus revealing its potential role as a change-of-function mutation in the ORF7a protein. This conformational stability is different from the one commonly reported through RMSF comparative evaluation. Inter-simulation per-residue average RMSF plots are routinely used to compare the flexibility of protein loops, with good results obtained on viral proteins, such as SARS-CoV-2 main protease monomer vs. dimer [30] and WT vs. mutant [31] or spike protein Receptor-Binding Domain (RBD) of SARS-CoV vs. SARS-CoV-2 [32]. In these prototypic examples, differential per-residue average RMSF among simulated molecules reflects changes in ligand−protein or protein−protein binding abilities due to changes in loop flexibility, i.e., the fluctuation of atomic coordinates over time. Average RMSF from several replicas are typically calculated, given that MD replicas behave differently from one simulation to another due to short, simulated time scales and the MD algorithmic assignment of different initial atomic velocities. In our case, we reveal almost identical per-residue average RMSF values (i.e., no changes in flexibility), yet the simulations of the mutant, disease-enhancing protein show little RMSF variation among the six replicas in specific regions. The alpha carbons of most residues in the 21–71 region fluctuate to the same degree over time (in respect to the controls) from their average coordinates, but they seem to fluctuate with minimal variation in the six replicas (unlike the controls). We contend that the mutation imprints, to an unusually large degree, a deterministic conformational behavior of the protein, which undergoes very specific and stable fluctuation patterns, in solvent-exposed loops. To identify the residues that undergo changes in the A105V simulation, we performed pair-wise F-tests for variance. Most residues in the 25–58 range had very low *p*-values, thus rejecting the null hypothesis of equal RMSF variance for most amino acids placed in that region (Figure 4A). This was reflected in the “A105V vs. WT” analysis (Figure 4A, blue line), but also when comparing A105V to A106V simulations (Figure 4A, red line). However, this was not observed when comparing A106V to WT simulations (Figure 4A, green line). A structural overview of the per-residue *p*-value shows low *p*-values in the core of the protein, as expected (Figure 4B). Notably, most of these residues were found in the beta-sheet formed by the 39–56 strand, but also in the adjacent 26–30 loop (Figure 4C), 17 of which were statistically different when comparing A105V vs. WT (*p* < 0.05, F-test; Figure 4C).

The leucine residue in position 102 that is in direct contact with the residue from position 105 was shown to be the most statistically significant (*p* = 0.018), suggesting that the effect of our mutation of interest is very likely due to direct interactions between the residues in positions 102 and 105. Indeed, the hydrophobic van der Waals interaction energy between residues in positions 102 and 105 was stronger for L102-V105 compared to L102-A105 (Figure 4D). This particular interaction exerts its effects through allosteric mechanisms, which are mainly transduced through dynamic changes of two solvent-accessible strands formed by residues 26–30 and 39–56. Due to their location, these residues are in direct contact with potential interaction partners of ORF7a.

## 4. Discussion

The current COVID-19 pandemic requires in-depth investigation of the molecular mechanisms of its causative agent, the novel SARS-CoV-2, in order to address therapeutic potential and understand the epidemiological context. In this study, we applied computational analyses via molecular dynamics simulations of SARS-CoV-2 accessory protein ORF7a mutation, A105V, which was identified as being responsible for the severe clinical form of COVID-19 disease in a set of Romanian patients, suggesting a potential gain-of-function role of this protein [21]. Our clinical data showed that, compared to controls, patients infected with A105V mutation developed a severe form of the disease, which was associated with higher systemic inflammation and increased damage to the lungs. In addition, A105V-infected patients had a lower viral load and increased death rates despite presenting none or fewer morbidities compared to patients lacking this mutation, suggesting increased virulence of A105V mutation.

**Figure 5:**
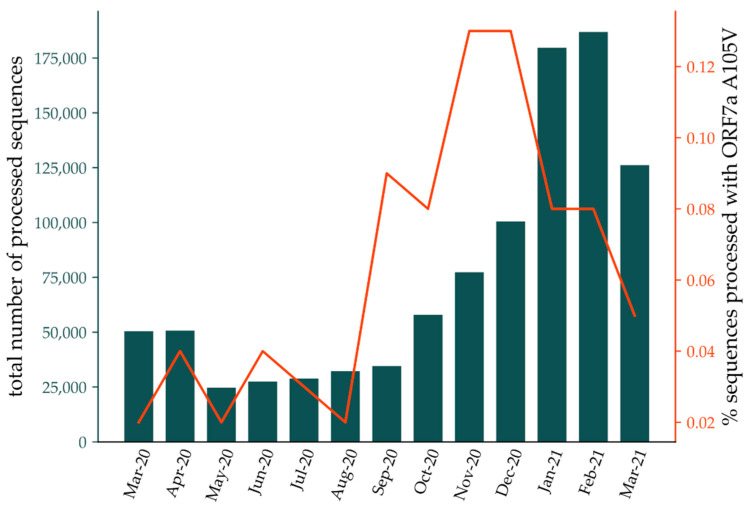
Rate of ORF7a A105V mutation in sequences processed by CoV-GLUE [33] (orange line) and total number of sequences processed by the server (dark green bars) between March 2020 and March 2021.

MD analyses (per-residue RMSF) on WT, mutant A105V, and mutant A106V showed that the A105V mutation alters the protein in a specific way, changing the conformational landscape and likely influencing its atomistic interaction potential. We posit that the mutation alters the binding affinity of ORF7a to one of its interaction partners, pointing to the overall importance of this protein in SARS-CoV-2 infection severity. These findings corroborate with generally low, but highly variable rates of ORF7a A105V mutations in overall worldwide sequences processed by CoV-GLUE platform (Figure 5) [33], which amounted to 0.12% of all sequences in late 2020. This low mutation rate confirms our observations of a high disease severity and increased lethality, which correlate with low transmissibility. The high variation rates are expected, due to the increased susceptibility to mutations of ORF7a gene [17].

When considering the drivers of this specific mutation, one has to take into account mutational patterns already observed in SARS-CoV-2, with the highest number occurring in ORF proteins. Such proteins are involved in disrupting host immune mechanisms, thus, their frequent mutations could aid the virus in escaping defenses. In particular, ORF7a shows a high susceptibility to mutations, and these mutations occur more frequently towards the C-terminus, as shown in a recent surveillance report [17]. Truncation of the C-terminus, including ablation of the transmembrane domain, negates anti-immune activities of the protein, suggesting a critical role for the transmembrane domain in ORF7a protein pathogenic function. While conservation of functional regions (i.e., protein−protein interaction domains) is opportune and leads to a purifying selection mechanism in some regions, ORF7a mutations and the advantages they confer to the virus could be driven by a positive selection pressure. Nevertheless, an enormous number of host−pathogen interactions occur, within different or even the same virus population, and probably more advantageous ones can supersede others. Additionally, ORF7a variants seem to occur spontaneously and independently in various geographic regions, and, at the same time, ORF7a mutants come from genomes forming monophyletic clades. The latter suggests that ORF7a mutations can propagate within a host population and that future genomic surveillance analyses should take into account the identified gain-of-function mutation. Considering all factors (the genomic heterogeneity of our sample, the unpredictability occurring of ORF7a mutations, the high mutation rates, the potential of ORF7a mutations to form monophyletic clades), our results can be extrapolated to other novel occurrences of mutations substituting the alanine residue in position 105, or its interacting residue, L102. The clinical evolution of patients identified with mutations in these regions should also be closely monitored.

ORF7a binds to several cellular receptors and interactions partners, and we hypothesize that A105V mutation translates in an ORF7a protein with differential binding affinities, which may lead to a different interaction landscape and more adverse clinical outcome. For example, ORF7a of SARS-CoV binds to Bcl-X_L_ and other related prosurvival proteins and thus may inhibit their anti-apoptotic role. The transmembrane domain of ORF7a is needed for this interaction, and it is likely that this interaction occurs with the transmembrane domain of Bcl-X_L_ [34]. Another interacting partner of SARS-CoV ORF7a is human BST-2. This protein is mainly localized on membranes of type I interferon producing cells, and its expression is largely induced in other cell types through type I interferon treatment. ORF7a has been shown to interfere with BST-2 function through a completely novel mechanism, leading to direct protein−protein binding to BST-2 and subsequent inhibition of its glycosylation at positions 65 and 92. SARS-CoV ORF7a binds unglycosylated BST-2 with higher affinity than N-linked glycosylated BST-2 [35].

Despite past and recent advances on ORF7a and its interaction potentials in human cells, the role of this accessory protein in SARS-CoV-2 infection remains to be fully elucidated [35]. However, recent studies show a pivotal role of this protein in SARS-CoV-2 mechanisms of virulence. SARS-CoV-2 ORF7a binds to receptors expressed on CD14+ monocytes in human peripheral blood, to alter, in a negative way, the immune response [11]. It may also bind to T lymphocytes and macrophages [12] and has also been linked to viral transmission and pathogenesis through inhibition of interferon signaling [13,14]. Transcription of antiviral IFN-stimulated genes (ISGs), which act to impede viral replication, is blocked by SARS-CoV-2 ORF7a through suppressing phosphorylation of STAT2 [13,36]. The ability of SARS-CoV-2 ORF7a to antagonize IFN-I responses is mediated by hijacking the host ubiquitin system to polyubiquitinate ORF7a [14]. An MD-based computational study performed by Ongaro and coworkers [37] revealed multiple stable binding conformations between SARS-CoV-2 ORF7a and lymphocyte function-associated antigen 1 (LFA-1). Finally, a recent study showed that ORF7a also interacts with ribosomal transport protein HEATDR3 and MDN1, a pathway whose role in pathogenesis or drug discovery is not known [2,15] but begs the need for further progress on SARS-CoV-2 ORF7a-associated pathogenesis mechanisms.

In agreement with other studies, our work confirms the suitability of ORF7a models in molecular dynamics analyses and related methods [37]. Retrieving the structural flexibility of a protein using the RMSF as an indicator is a widely used practice and has been applied in MD simulations of various SARS-CoV-2 proteins [30,32,38]. Residue-by-residue RMSF analysis is a common and powerful used method for evaluating protein-related phenomena, ranging from protein evolutionary changes [39] to residue or domain flexibility changes due to mutations [40,41] or temperature [42]. The common approach is to employ comparisons of average RMSF among the studied proteins, which then reveal the protein domains that undergo flexibility changes that translate into changed protein function. However, in our particular case, since the average RMSF was relatively identical among the three protein forms (Figure 3B), we examined differences based on the RMSF’s standard deviations. The RMSF standard deviation of A105V simulations in the protein core residues was low, reflecting a high structural stability that is suggestive of a highly deterministic behavior and a subtle allosteric effect of the mutation. We showed that the computational approach based on clinical data, combined with in-depth RMSF variance analysis, can yield significant results that can be overlooked by traditional RMSF-based analyses. By employing this model, we also avoided multiple short replicas that can lead to variations in correctly identifying positives in MD-based investigations. F-tests for variance were carried out in order to pinpoint the residues involved in transducing the observed effects. The beta-sheet placed at the exterior of the protein core is formed by residues in position 39–56, but also the adjacent 26–30 loop, which undergo the lowest RMSF variance and are highly impacted through allosteric effects of A105V mutation. Such effects seem to manifest through the V105-L102 interaction, which then allosterically alters the overall protein stability at residues placed in direct contact with the solvent and, therefore, with ORF7a interaction partners. This may explain the behavior of A105V mutation in causing more severe clinical symptoms even in patients with fewer comorbidities and lower viral load.

## 5. Conclusions

In this study, we performed a computational structural MD-based investigation, guided by corroborating clinical data with SARS-CoV-2 RNA sequencing of COVID-19 patients. Analysis of the clinical and genomic data revealed a strong link between disease severity and mortality and a particular mutation in the ORF7a protein, A105V. Structural computational analyses showed that A105V changed the conformational landscape most likely by altering the binding affinity of ORF7a protein. The study demonstrates the importance of ORF7a mutation A105V as a gain-of-function protein and paves the way for further work in determining its precise biological impact at cellular level that plays an important role in COVID-19 pathogenesis.

## Figures and Tables

**Table 1:** Summary of COVID-19 patients’ data.

		ORF7a WT		ORF7a A105V	
		Values ^1^	Patients	Values	Patients
General data	Age (years)	53.24 ± 2.88	(*n* = 45)	50.06 ± 4.21	(*n* = 17)
	Sex (%)	37.78 F 62.22 M	(*n* = 45)	35.29 F 64.71 M	(*n* = 17)
PCR data	CT (N)	23.75 ± 0.71	(*n* = 40)	25.36 ± 0.97	(*n* = 12)
	CT (S)	24.9 ± 0.62	(*n* = 41)	26.91 ± 0.9	(*n* = 13)
	CT (R)	26.27 ± 0.65	(*n* = 34)	26.7 ± 0.92	(*n* = 16)
Blood tests	Leucocytes (cells × 10^9^/L)	8.76 ± 1.02	(*n* = 33)	10.03 ± 2	(*n* = 8)
	CRP (mg/dL)	5.59 ± 1.17	(*n* = 35)	9.66 ± 2.7 *	(*n* = 15)
	Thrombocytes (cells × 10^3^/µL)	272.22 ± 31.61	(*n* = 23)	197.5 ± 29.9	(*n* = 6)
	Hemoglobin (g/dL)	12.56 ± 0.44	(*n* = 30)	12.2 ± 0.74	(*n* = 10)
	ALAT/GPT (U/L)	55.11 ± 7.29	(*n* = 27)	47.24 ± 9.32	(*n* = 10)
Comorbidities (%)	Hypertension	42.50	(*n* = 40)	21.43	(*n* = 14)
	Obesity	37.50		21.43	
	Diabetes	17.50		14.29	
	Min. 1 comorbidity	50.00		35.71	
Other clinical data	Days in hospital	20.27 ± 1.57	(*n* = 33)	20.33 ± 2.45	(*n* = 12)
	Deaths due to COVID-19 (%)	9.52	(*n* = 45)	18.75	(*n* = 17)

^1^ Values are means ± SEM. Abbreviations: CT, cycle threshold; CRP, C reactive protein; ALAT, alanine aminotransferase (a.k.a. GPT, glutamic-pyruvic transaminase); Ct, qPCR cycle threshold; N, nucleocapsid; S, spike; R, RNA-dependent RNA polymerase (RdRp); * statistically different (*p* < 0.05, Student’s *t* test).

## Data Availability

The data presented in this study are available on request from the corresponding author. The data are not publicly available due to patient confidentiality.

## Supplementary Materials

The following are available online at https://www.mdpi.com/article/10.3390/biology10121276/s1. Figure S1: Chimera-enabled spatial rearrangement of ORF7a transmembrane helix.
