# Supplementary material for: A Conservative Replacement in the Transmembrane Domain of SARS-CoV-2 ORF7a as a Putative Risk Factor in COVID-19
Source: Biology (Basel). 2021 Dec 5;10(12):1276. doi: 10.3390/biology10121276 (PMC8698902; doi:10.3390/biology10121276)
Supplement: Supplementary file 1 [file biology-10-01276-s001.zip › biology-1481433-supplementary.pdf]

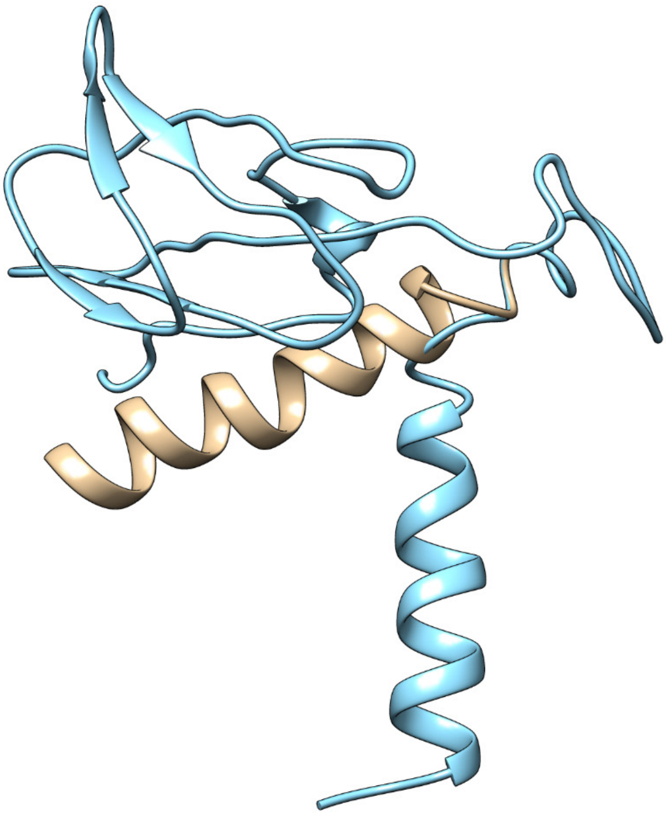

Figure S1. Chimera-enabled spatial rearrangement of ORF7a transmembrane helix. The spatial arrangement is shown prior (light brown) and after (light blue) Chimera-enabled transformations.
